# Brain-Derived Neurotrophic Factor Levels in Cannabis Use Disorders - A Systematic Review and Meta-Analysis

**DOI:** 10.7759/cureus.45960

**Published:** 2023-09-25

**Authors:** Palani S Mohanraj, Arani Das, Aniruddha Sen, Manoj Prithviraj

**Affiliations:** 1 Biochemistry, All India Institute of Medical Sciences, Gorakhpur, IND; 2 Physiology, All India Institute of Medical Sciences, Gorakhpur, IND; 3 Psychiatry, All India Institute of Medical Sciences, Gorakhpur, IND

**Keywords:** systematic review, meta-analysis, cannabis, tetrahydrocannabinol, neurotrophins, marijuana, substance abuse, brain-derived neurotrophic factor, cannabinoids

## Abstract

The prevalence of cannabis use disorders has become a noteworthy global public health issue. Understanding the neurobiological factors associated with cannabis use disorder (CUD) is crucial for creating effective interventions. One such factor, the brain-derived neurotrophic factor (BDNF), has been linked to the onset and persistence of addictive behaviors. This systematic review aims to summarize the existing literature on BDNF levels in individuals with CUD to provide a comprehensive overview of the current evidence. A systematic search was conducted using electronic databases (PubMed, Scopus) for relevant studies. The search approach yielded a total of 785 articles, with 559 located in the PubMed database and 226 in Scopus. Studies reporting BDNF levels in individuals with CUD compared to healthy controls were included in this study. Ultimately, eight articles were included in this systematic review. The primary emphasis of these studies was on individuals who were cannabis users or had a dependency on cannabis. There is considerable variation in the estimated effect size among included studies due to heterogeneity; hence, a random effect model was used for meta-analysis. The findings of our study suggest that the effect size of BDNF levels was 0.25 with 95% CI (-0.55; 1.05) in cannabis users, which was not statistically significant (p-value=0.54). Therefore, it is important to interpret the results with caution, and additional research is warranted to investigate the potential factors contributing to this heterogeneity.

## Introduction and background

Cannabis is probably the oldest psychoactive substance known by mankind. Most of the psychoactive effects are believed to be due to a principal compound, tetrahydrocannabinol (THC). Effects of THC are mediated by cannabinoid receptor 1 (CB1) and cannabinoid receptor 2 (CB2), distributed in different parts of the brain, together forming an endocannabinoid system [1]. Studies have linked chronic exposure to cannabis with persistent cognitive deficits [2,3]. Further, exposure to cannabinoids in the adolescent and perinatal periods is associated with a higher risk of schizophrenia, psychosis, and substance use disorders [4].

Few studies have shown that long-term use of cannabis could lead to changes in brain health, including changes in neuronal and axonal integrity and reductions in brain volume in specific regions like the parahippocampal gyrus and parietal lobe [5,6]. Brain-derived neurotrophic factor (BDNF) plays a crucial role in supporting the development, maintenance, and survival of neurons [7]. BDNF is also involved in synaptic plasticity and normal functioning of dopaminergic and cholinergic neurons in the midbrain [8,9]. Dysfunctions in the production and utilization of neurotrophins have been associated with several central nervous system (CNS) disorders [10]. Few experimental studies suggest that psychoactive substances can alter neurotrophin levels [11]. For example, BDNF expression in specific areas of rat brains increases after exposure to amphetamine, a dopamine agonist [12]. Withdrawal from cocaine use has also been shown to increase BDNF levels in certain brain regions of rats, suggesting a potential role of BDNF in substance addiction [13]. Similarly, chronic amphetamine use is associated with reduced synthesis of nerve growth factor (NGF) and BDNF in the rat brain [14]. Chronic heroin and cocaine abuse in humans is found to decrease serum levels of NGF and BDNF [15]. This evidence suggests that reduced neurotrophin production could contribute to the development of psychiatric disorders in cannabis-dependent individuals.

Several studies have tested this hypothesis by measuring serum BDNF levels in cannabis users with variable results [10,11,16-21]. Therefore, the aim of this systematic review is to investigate and summarize the existing literature regarding the levels of BDNF in individuals with cannabis use disorder. 

## Review

Materials and methods

The systematic review adhered to the Preferred Reporting Items for Systematic Reviews and Meta-Analyses (PRISMA) guidelines, which provide a framework for reporting systematic reviews and meta-analyses [22].

Selection Procedure

The systematic review focused on the relationship between cannabis use and serum BDNF levels. Participants of any age and gender with a history of any form of cannabis use were included in the review. The review excluded participants with any comorbid physical and mental health conditions. Any form of cannabis use was considered, with no restriction to dosage form, frequency, and dose. Healthy volunteers without a history of cannabis use served as the comparator/control. Observational cross-sectional studies that had assessed the levels of BDNF among individuals with cannabis use were included. The main outcome was serum BDNF levels. The protocol for the review was registered on PROSPERO, and it was assigned a registration ID: CRD42022379533.

Search Strategy

We conducted a systematic search of the literature in PubMed and Scopus from the date of their inception to September 2022. Additionally, we searched the bibliographies of relevant research articles. We only included studies written in the English language. The following search strategy was utilized (Table 1).

**Table 1:** Search strategy

Database	Search terms
Pubmed	("cannabis"[MeSH Terms] OR "cannabis"[All Fields] OR"tetrahydrocannabinol"[All Fields] OR "tetrahydrocannabinols"[All Fields] OR "marijuana"[All Fields] OR "marijuana s"[All Fields] OR ("cannabinoids"[MeSH Terms] OR "cannabinoids"[All Fields] OR "cannabinoid"[All Fields]) OR ("cannabis"[MeSH Terms] OR "cannabis"[All Fields] OR "cannabi"[All Fields] OR "cannabis s"[All Fields])) AND ("brain derived neurotrophic factor"[MeSH Terms] OR ("brain derived"[All Fields] AND "neurotrophic"[All Fields] AND "factor"[All Fields]) OR "brain derived neurotrophic factor"[All Fields] OR "bdnf"[All Fields])
Scopus	TITLE-ABS-KEY (("cannabis" OR "tetrahydrocannabinol" OR "tetrahydrocannabinols" OR "marijuana" OR "marijuana s" OR "cannabinoids" OR "cannabinoid" OR "cannabis" OR "cannabi" OR "cannabis s") AND ("brain derived neurotrophic factor" OR ("brain derived" AND "neurotrophic" AND "factor") OR "brain derived neurotrophic factor" OR "bdnf"))

Screening and Data Analysis

Studies were screened on the basis of title and abstract, and full text of relevant studies full were retrieved for screening by two independent reviewers using an open access online tool CADIMA version 2.2.3 (Julius Kühn Institute, Quedlinburg, German). Any discrepancies were resolved by discussion or consulting with a third researcher. Two independent reviewers performed data extraction from the selected studies using a standardized form, and any discrepancies were resolved by consensus or consultation with another reviewer. Data extraction included participant demographics (age, gender, details of cannabis use), sample characteristics (country, sample size), exposure characteristics (cannabis use, age of onset, duration of use, form, frequency, and dose), study characteristics (design of the study, BDNF levels in serum), and was recorded in an excel spreadsheet. The data were analyzed using R statistical software using the 'meta' package (R Foundation, Vienna, Austria). For continuous variables such as BDNF levels, results were pooled and expressed as mean differences (MD) based on inverse variance methods with 95% confidence intervals (CI). Heterogeneity among studies was assessed using Cochran's Q and I² statistics. Based on heterogeneity, a fixed effect or random effect model was used appropriately.

Quality Assessment

**Figure 1:**
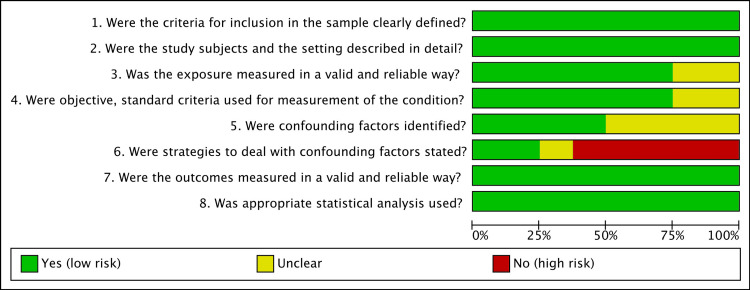
Risk of bias graph Risk of bias was assessed using the critical appraisal checklist for cross-sectional studies by the Joanna Briggs Institute [23]

**Figure 2:**
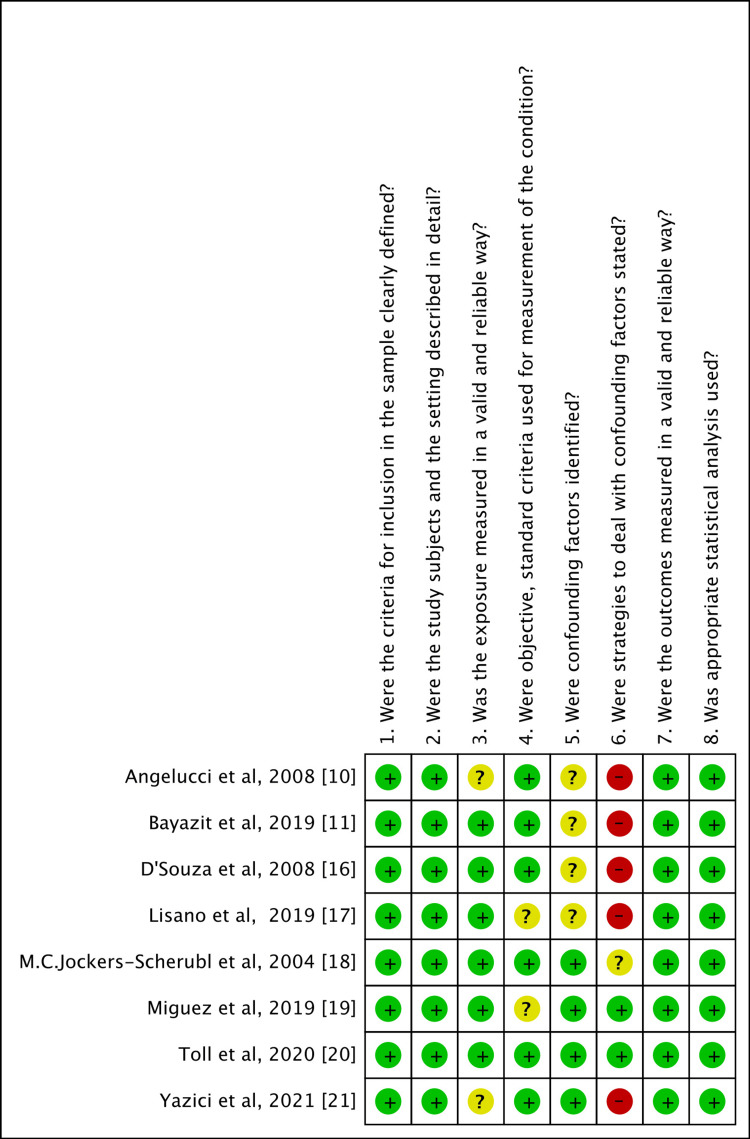
Risk of bias summary Risk of Bias was assessed using the critical appraisal checklist for cross-sectional studies by the Joanna Briggs Institute [23]

The quality and the presence of biases in the study included in the systematic review was assessed using the critical appraisal checklist for cross-sectional studies by the Joanna Briggs Institute [23]. The risk of bias summary and graph was created using RevMan 5.4 (Revman International, Inc., New York City, New York) (Figure 1, 2).

Study Characteristics

**Figure 3:**
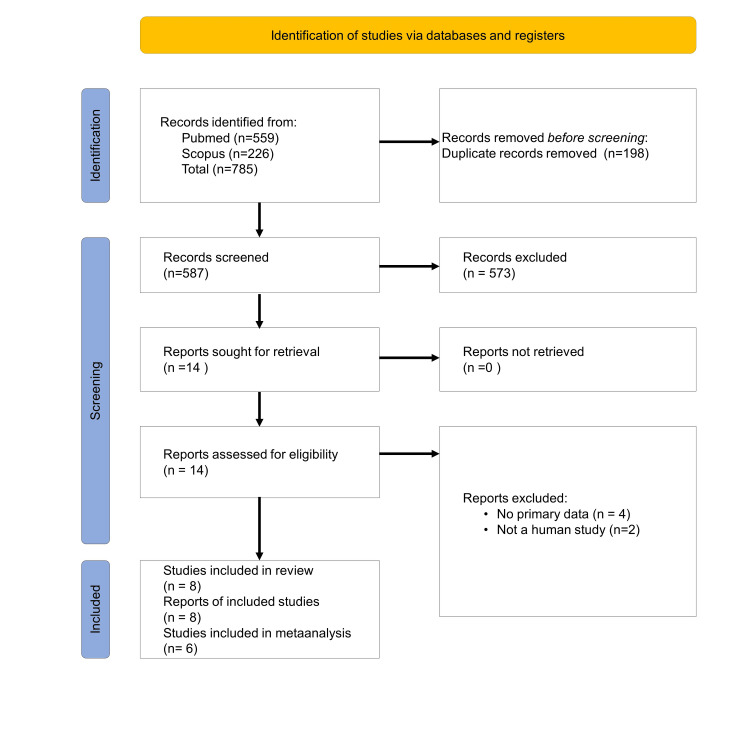
PRISMA flow diagram PRISMA - Preferred Reporting Items for Systematic Reviews and Meta-Analyses

The search strategy found 785 articles, 559 of which were in the PubMed database and 226 in Scopus. After removing duplicates and merging results from all databases, a total of 587 articles were identified. The PRISMA flow chart of the search process is depicted in Figure 3.

After screening the title and abstract, 14 articles that were eligible for full-text screening, six were excluded due to reasons listed in Figure 3. Finally, a total of eight articles were included in this systematic review. Among them six articles were included for meta-analysis. Two articles were excluded because of non-availability of control BDNF level in one and BDNF level was expressed as median with interquartile range (IQR) in another [16,20].

The studies included in the systematic review were conducted in five different countries: USA, Italy, Germany, Spain, and Turkey. The studies mainly focused on cannabis users/dependent subjects and the sample size of the included studies ranged from 23 to 490 subjects. Basic characteristics of the included studies along with details of intervention and study outcome are given in Table 2, 3.

**Table 2:** Basic characteristics of included studies *Data are presented as mean ± SD or median (range) E - exposure group (cannabis users); C - comparator group (controls)

Study	Country	Population	Cannabis use	Sample size	Mean age, years	Gender distribution	Design
Angelucci et al., 2008 [10]	Italy	Cannabis users	Age of onset: 16.5 ± 2.65 years; Duration: 10.73 ± 5.22 years	E=26; C=20	E: 27.3 ± 5.64; C: 28.25+_4.84	E: 14M/12F; C: 10M/10F	Cross-sectional observational study
Bayazit et al., 2020 [11]	USA	Cannabis dependent	Duration: 69 ± 7 months	E=45; C=45	E: 25 ± 7; C: 25 ± 8	-	Cross-sectional observational study
D'Souza et al., 2009 [16]	USA	Light cannabis user	Light user of cannabis not defined properly	E=9; C=14	E=22.66 ± 2.82; C: 25.85 ± 7.98	E: 9M; C: 11M/3F	Interventional study
Lisano et al., 2020 [17]	USA	Cannabis users	Age of onset: 16.33 ± 1.84 years; duration: 6.87 ± 3.94 years; uhronic users: once per week for last 6 months	E=15; C=15	E: 23.2 ± 3.7 C: 23.5 ± 5.1	E: 10M/5F; C: 10M/5F	Cross-sectional observational study
Jockers-Scherubl et al., 2004 [18]	German	Cannabis users	>0.5g of cannabis per day for >2 years	E= 11 ; C=61	E: 29.6; C: 32.3	E: 9M/2F; C: 28M/33F	Cross-sectional observational study
Miguez et al., 2019 [19]	USA	Marijuana users	-	E=152; C=338	E: 16.5 ± 1.4; C: 14.5 ± 2.2	E: 68 M/84F; C: 162M/176F	Longitudinal cohort study follow up duration 12 months
Toll et al., 2020 [20]	Spain	Cannabis users	Low user: no detail; High User: no detail; age of onset:15.76 ± 3.11 years; duration: 4.58 ± 2.53 Years	Low E=19; high E=14; C=24	Low E=22 (20.79-24.58); High E=20 (18.44-24.71); C=24 =23.5 (22.5-26.31)	43M/14F	Cross-sectional observational study
Yazici et al., 2022 [21]	Turkey	Chronic cannabis user	Age of onset: 19.07 ± 5.98; duration: 9.61 ± 4.76 years	E=27; C=27	E:29.62 ± 6.12; C:30.70 ± 7.05	-	Cross-sectional observational study

**Table 3:** Details of intervention used and Study outcome E - exposure group (cannabis users); C - comparator group (controls); BDNF - brain-derived neurotrophic factor

Study	Comparison group	BDNF level	outcome
Angelucci et al., 2008 [10]	Healthy controls	E: 5984.23 ± 335.9 pg/ml; C: 5683.62 ± 237.65 pg/ml	No significant difference in cases and controls
Bayazit et al., 2020 [11]	Healthy controls	E: 74.79 ± 13 pg/ml; C: 55.27 ± 12.5 pg/ml	BDNF increased, Significant difference
D'Souza et al., 2009 [16]	Healthy controls not well defined, previous cannabis users were used as comparator group	-	Cannabis users has low BDNF levels
Lisano et al., 2020 [17]	Healthy controls	E: 5.61 ± 0.78 ng/ml; C: 6.3 ± 0.8 ng/ml	BDNF low, significant difference
Jockers-Scherubl et al., 2004 [18]	Healthy controls	E:13.1 ± 2.7ng/ml; C: 13.2 ± 5.2ng/ml	No significant difference in cases and controls
Miguez et al., 2019 [19]	Healthy controls	E: 3731.1 ± 903.4 pg/ml; C: 2046.2 ± 262.5 pg/ml	High BDNF among marijuana users compared to non-users after 12 months longitudinal study
Toll et al., 2020 [20]	Healthy controls	C: 308 (216.39– 369.52) pg/ml; low E: 174 (172.21– 326.64) pg/ml; high E:186 (124.84– 387.45) pg/ml	No significant difference in cases and controls
Yazici et al., 2022 [21]	Healthy controls	E:4.28 ± 5.23 ng/ml; C:4.83 ± 4.47 ng/ml	No significant difference in cases and controls

The main outcome of the included studies was the serum BDNF levels. Out of the eight studies included in this review, six studies had serum BDNF level data available as mean and standard deviation and were included for meta-analysis. One study was excluded from the meta-analysis due to non-availability of BDNF values. Another study was not included in the meta-analysis due to serum BDNF levels expressed as median and IQR. This meta-analysis aimed to synthesize the results of six studies investigating the effect of cannabis use on serum BDNF levels. The meta-analysis employed both the fixed-effect model and the random-effects model to estimate the common effect size and to quantify the heterogeneity among the studies. A summary of the meta-analysis is given in Table 4.

**Table 4:** Summary of meta-analysis

Study	MD	95%-CI	%W (common)	%W (random)
Angelucci et al., 2008 [10]	0.3000	0.1380; 0.4620	0.1	21.5
Bayazit et al., 2020 [11]	0.0190	0.0138; 0.0242	99.8	21.7
Lisano et al., 2020 [17]	-0.6900	-1.2554; -0.1246	0.0	19.6
Jockers-Scherubl et al., 2004 [18]	-0.1000	-2.1612; 1.9612	0.0	8.9
Miguez et al., 2019 [19]	1.6850	1.5388; 1.8312	0.1	21.6
Yazici et al., 2022 [21]	-0.5500	-3.1451; 2.0451	0.0	6.7

**Figure 4:**
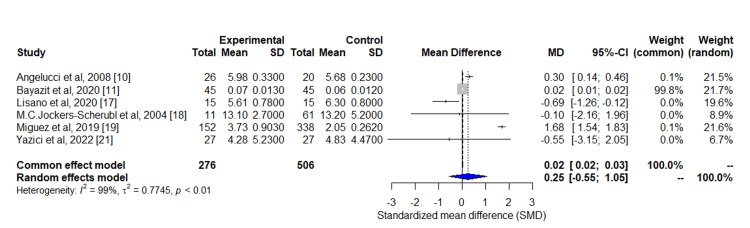
Forest Plot Forest plot of the included studies: number of studies: k = 6; number of observations: o = 782; quantifying heterogeneity: tau^2 ^= 0.7745 (0.2142; 4.1856); tau = 0.8801 (0.4628; 2.0459); I^2 ^= 99.0% (98.7%; 99.3%); H = 10.15 (8.69; 11.87); test of heterogeneity: Q=515.5; degree of freedom= 5; p-value<0.0001; details on meta-analytical method: inverse variance method, restricted maximum-likelihood estimator for tau^2^, Q-Profile method for confidence interval of tau^2 ^and tau

The common effect size, represented by the mean difference (MD), was estimated to be 0.02 with a 95% confidence interval (CI) of (0.02; 0.03; p<0.0001) using the fixed-effect model. The test for heterogeneity indicated significant heterogeneity among the studies (Q=515.5, p<0.0001), and the random-effects model estimated the MD to be 0.25 with a wider 95% CI of -0.55; 1.05 (p=0.54). The heterogeneity was further quantified by estimating the tau-squared (τ^2) to be 0.7745 with a 95% CI of 0.2142; 4.1856, which indicates a considerable amount of heterogeneity. The I^2^ value of 99.0% and the Higgins' H statistic of 10.15 (95% CI: 8.69; 11.87) also suggest substantial heterogeneity. A forest plot of the included studies is given in Figure 4.

The sensitivity analysis also revealed variations in this combined effect size when omitting each study one by one. Most notably, when Miguez et al.'s study [19] was omitted, the MD was significantly lower. Given the evident heterogeneity, a random-effects model, which accounts for inter-study variability, may offer a more conservative and appropriate effect estimate in the face of heterogeneity.

Although there is a significant effect of cannabis use on serum BDNF levels based on the fixed effect model, but the effect size estimate varies substantially across different studies due to the heterogeneity. Therefore, caution should be taken when interpreting the results, and further research may be needed to explore the potential sources of heterogeneity.

Discussion

BDNF has been implicated in various psychiatric disorders. In this study, we aimed to investigate whether cannabis use has any association with alteration in serum levels of BDNF. We found eight studies where serum BDNF level was measured among cannabis users. Among them, four case-control studies have shown no significant change in the serum BDNF level, two showed an increase, and two studies reported decreased serum BDNF levels between cannabis users and respective controls. For meta-analysis, we have included data from six studies.

Cannabidiol (CBD), a non-psychoactive component of cannabis, has been shown in some studies to increase BDNF levels, perhaps giving neuroprotective advantages [24]. Increased BDNF levels have been associated with enhanced neuroprotection, neurogenesis, and improved antioxidant defense systems in the brain, helping neurons overcome oxidative stress and maintain cellular integrity [25]. This neuroprotective effect of BDNF has been suggested to have therapeutic implications for various neurodegenerative diseases and conditions associated with oxidative stress [26]. These investigations, however, are mostly conducted on animals or in small-scale human trials, and further study is needed to corroborate these findings.

THC, the psychoactive component in cannabis known for the "high," on the other hand, has been linked to deleterious effects on cognitive function and memory [27]. Multiple studies have reported a wide range of CNS effects on the administration of THC [28,29]. For example, the administration of THC activates the cannabinoid receptor CB-1R, leading to an increase in mesolimbic dopaminergic activity, which provides an explanation for the positive psychotic symptoms in rats [30]. Exposure of THC in humans also produces psychotic symptoms resembling the effects of dopamine agonists [31]. So primarily, cannabis produces agonist action on dopaminergic neurons, which may lead to deranged neurotrophin levels and also may contribute to the pathogenesis of psychiatric disorders. These studies hypothesize that the consumption of cannabis reduces brain neurotrophins, which leads to neurotoxicity and potentially gives rise to drug-related psychiatric disturbances. In a study by Angelucci et al., they have reported chronic amphetamine treatment also reduces NGF and BDNF in the rat brain [14].

Cannabis use may have harmful effects, especially in people with developing brains (such as adolescents) and those with a history of mental health disorders. While acute cannabis usage can alter serum BDNF levels, the findings have been inconsistent. Some studies have found a drop in BDNF levels after acute THC exposure, which may be connected to the impairments in learning and memory that are typically noted after cannabis use [17]. Other research investigations, however, have revealed that THC has a biphasic effect on BDNF levels, with low to moderate dosages increasing BDNF levels and high doses decreasing them [16]. Nonetheless, this response does not appear to be constant across all experiments. The effect of cannabis on BDNF levels is complex and varies based on dosage, duration of usage, individual variances, and the precise composition of the cannabis product.

While this review provides valuable insights, several limitations should be acknowledged. The sensitivity analysis shows that the study by Miguez et al. [19] stands out as having a profound influence on the overall result. The variance in MDs upon the sequential omission of studies suggests potential heterogeneity. Further investigations, such as subgroup analyses or meta-regression, might be helpful to pinpoint the sources of heterogeneity. The exclusive reliance on cohort studies available in the literature warrants the need for future research endeavors with larger sample sizes to provide a more comprehensive understanding of the subject matter.

## Conclusions

In conclusion, our meta-analysis sheds light on the relationship between cannabis use and BDNF levels. While our study results suggest increasing BDNF levels among cannabis users, the difference isn't statistically significant. It's essential to delve deeper into this relationship to fully grasp cannabis's long-term effects on brain and mental health. As we consider cannabis for therapeutic purposes, it's imperative to educate stakeholders about potential risks and the importance of moderation.
